# Obstetrics, and Diseases of Women and Children

**Published:** 1859-03

**Authors:** 


### OBSTETRICS, AND DISEASES OF WOMEN AND CHILDREN.

IX.—1. *Death after an Injection of Carbonic Acid into the Uterus.* By PROFESSOR SCANZONI. 2. *Experiments with Carbonic Acid on Pregnant Rabbits.* By DRS. BRESLAU and VOGEL.

THE woman whose case Professor Scanzoni reports in the third volume of his *Beiträge zur Geburtskunde und Gynäkologie,* (p. 181,) 1858, was pregnant, but in consequence of an error in diagnosis, amputation of the hypertrophied cervix uteri was decided upon; it protruded out of the vulva in the form of a bluish, chinky tumor, of the size of a small apple. The attending physician, father of the patient, insisted upon having carbonic acid injected into the cavity of the uterus, preparatory to the operation, with the view to prevent too profuse a hemorrhage; this injection was made by means of a suitable apparatus; but hardly had some cubic inches of gas penetrated into the cervix, when the patient screamed that she felt the air rushing into her abdomen, her neck, and her head. General tetanic convulsions supervened; then a long agony. In about an hour and three-quarters the patient was a corpse.

On autopsy no other lesion was found to which death was attributable, but a very considerable œdema of the lungs. The womb contained an uninjured ovum of about four months.

This case excited considerable interest in Germany. The recommendation of Simpson and several other English physicians had induced obstetricians to make numerous experiments with carbonic acid—partly as a means to produce artificial labor, partly as local anæsthetic; and its efficacy had been proved by numerous facts. The method seemed to have recommended itself by its safety, although the experiments of M. C. Bernard, of Lyons, (*Archives Générales*, tome x. p. 529, 1857,) had already demonstrated that toxical symptoms may result from injections of carbonic acid into the vagina. These researches in reference to local anæsthesia serve to prove that absorption of the gas may produce cephalalgia, vertigo, stupefaction, debility, dimness of vision, nausea, and more or less profound somnolence; but these symptoms were not sufficiently serious to give reason to fear the possibility of a fatal termination, and the report of the case of Professor Scanzoni was read everywhere with painful surprise.

Is this unforeseen death to be attributed to poisoning qualities of carbonic acid? or could the gas have penetrated into the orifice of an open vein, and have acted like air introduced into the veins? Professor Scanzoni himself remained in doubt about these questions, and it was with a view of throwing light upon them that Dr. B. Breslau and A. Vogel, of Munich, made a series of experiments on female rabbits in the different stages of pregnancy and puerperal state. The results of these experiments were as follows:—

The even prolonged injection of a great quantity of carbonic acid into the vagina or the peritoneum of pregnant rabbits, exercised no unfavorable influence upon their general health, nor upon the life of the fœtus; gestation suffered no modification from abundant intra-vaginal injections of even half an hour's duration. In making the injection under high pressure into the vagina or uterus, eighteen hours after delivery, no bad symptom was produced; although it is probable that the uterus presented at this period still some open vascular orifices.—(*Wiener Medizinische Wochenschrift*, September 11, 1858.)

X.—*On the Treatment of Syphilis in Pregnant Women.* By M. E. BERTIN.

Among the facts which have contributed the most to make the employment of mercury during pregnancy objectionable, and to diffuse the opinion that this medicine easily produces abortus or death of the fœtus, those which M. Colson has recorded in his memoir, "De l'influence du traitement mercuriel sur les fonctions de l'uterus," (*Archives Générales*, tome xviii. p. 24,) deserve especial notice. M. Bertin has subjected these not very numerous observations to a critical analysis, which shows peremptorily that not one of them has the demonstrative character which has been so readily attributed to them. He mentions then a remarkable case of Moriceau, which shows that mercury not only does not cause abortus, but that it may even prevent it, (Des maladies des femmes grosses, etc., 2d edit., p. 179,) and reports the history of eleven pregnant women who were treated in the *Maison de Secours* of Nancy. This is the complete series of all the cases which have presented themselves during the first four months of the year 1857.

All these patients, in whom pregnancy was more or less advanced, suffered from secondary symptoms, and were treated with pills of protiodide of mercury, or with the liquor of Van Swieten. Out of these eleven women, eight were delivered at term of living children, and their pregnancy had followed its natural course during their sojourn in the hospital; one of them was subjected to two mercurial treatments while she was pregnant, and did not experience any bad consequences. In the three other patients the pregnancy did not reach its term; but it is more than probable that the mercury had nothing to do with this result. In fact, one of these women was delivered of a dead child in a state of putrefaction, the movements of which had ceased to be felt before the mother entered the hospital. The second had miscarried already twice before she contracted the venereal disease, and it is possible the third abortus took place under the same influence as the two others. The last patient was delivered of a living child of seven months, upon which, consequently, the mercurial treatment could not have acted in a fatal manner.

M. Bertin concludes, from these facts, that mercury does not exert a fatal influence upon the human fœtus, (contrary to the opinion of Professor Trousseau,) and admits, with Ricord, "that the period of pregnancy, far from opposing the employment of energetic measures, demands still more attention and judicious promptitude."—(*Compte Rendu des Travaux de la Société de Médecine de Nancy*, p. 82, 1858, and *Gaz. Hebdomad.*, December 10, 1858.)

XI.—*Tubage of the Glottis by Forced Dilatation of the Larynx, in the Treatment of Croup.* By M. Bouchut.

In a memoir addressed to the *Académie de Médecine*, (September 14th,) M. Bouchut described a new method of treating croup, which consists essentially in introducing a silver tube into the larynx, through which air can more easily enter, and false membranes be expelled more readily. The canula is cylindrical, straight, from one to two centimetres long, and provided on its superior extremity with two round projections, placed at a distance of six millimetres from each other; it is provided with a hole through which a silk ligature is passed, designed to retain it. In introducing the instrument into the interior of the larynx, a curved probe, pierced at both extremities, serves as a conductor. It is placed into the glottis in such a manner that the two round projections, mentioned above, receive the inferior vocal chords into the space between them, (in a manner similar to a stud which confines the folds of a shirt bosom.) The tube is allowed to remain in place from one to three days, that is to say, until the phenomena of asphyxia have disappeared.

M. Bouchut has published two cases in his memoir, and a brief account of five others has been given in the report, read before the *Académie de Médecine*, by M. Trousseau.

The first case is that of a girl, five and a half years old, who was already cyanotic and suffocating when the apparatus was applied. The child seemed to be somewhat relieved by the canula, and the imminent asphyxia ceased. The patient succumbed, however, to repeated paroxysms. On autopsy, the existence of tubercular pneumonia was ascertained. There existed no traces of false membranes in the larynx and trachea; the mucous membrane was red, thick, and granular, on a level with the superior and inferior vocal chords, and on the interior surface of the cricoid. In the second case, that of a boy, three years and a half old, the operation was performed before the asphyxia had reached so high a degree, and was executed with great facility. Two large fragments of false membranes were ejected through the canula. In the absence of M. Bouchut, the respiration became embarrassed, and the child seemed in danger of suffocation; the "*internes*" of the hospital decided, therefore, to perform tracheotomy. According to the views of M. Bouchut, the necessity of the operation in this case could be called into question.

The author thinks these facts sufficient to show: 1. The facility with which tubage of the glottis can be executed by means of a canula fixed on the inferior vocal chords, and not hindering the functions of the epiglottis. 2. The tolerance of this tube by the larynx. 3. The possibility of remedying the asphyxia of croup and of the diseases of the larynx by this means, in preference to tracheotomy, a difficult and dangerous operation, the mortality of which varies from eighty to ninety per cent. 4. The facility with which the large pseudo-membranous concretions formed in the trachea and in the bronchi escape through this intra-glottic tube. 5. The utility of this new resource for physicians who live in the country, and are unable to procure the assistance necessary for the performance of other operations. The five other cases have not had a more favorable termination, and the tubage of the glottis has not yet been successful in averting the fatal end in a single instance.

The operation itself, its facility and advantages, are at present the object of an animated discussion before the *Académie de Médecine*, the result of which we will give at some future time.—*Gazette Hebdomad.*, and *Archives Générales*, November, 1858.)

